# Rare Germline *DICER1* Variants in Pediatric Patients With Cushing's Disease: What Is Their Role?

**DOI:** 10.3389/fendo.2020.00433

**Published:** 2020-07-03

**Authors:** Idoia Martínez de LaPiscina, Laura C. Hernández-Ramírez, Nancy Portillo, Ana L. Gómez-Gila, Inés Urrutia, Rosa Martínez-Salazar, Alejandro García-Castaño, Aníbal Aguayo, Itxaso Rica, Sonia Gaztambide, Fabio R. Faucz, Margaret F. Keil, Maya B. Lodish, Martha Quezado, Nathan Pankratz, Prashant Chittiboina, John Lane, Denise M. Kay, James L. Mills, Luis Castaño, Constantine A. Stratakis

**Affiliations:** ^1^Section on Endocrinology, Metabolism, Nutrition and Renal Diseases, Biocruces Bizkaia Health Research Institute, Cruces University Hospital, UPV/EHU, CIBERER, CIBERDEM, Barakaldo, Spain; ^2^Section on Endocrinology and Genetics, Eunice Kennedy Shriver National Institute of Child Health and Human Development (NICHD), National Institutes of Health (NIH), Bethesda, MD, United States; ^3^Pediatric Endocrinology Service, Alto Deba Hospital, Arrasate, Spain; ^4^Pediatric Endocrinology Service, Virgen del Rocío University Hospital, Sevilla, Spain; ^5^Pediatric Endocrinology Service, Cruces University Hospital, Barakaldo, Spain; ^6^Endocrinology Service, Cruces University Hospital, Barakaldo, Spain; ^7^Division of Pediatric Endocrinology, Department of Pediatrics, Mission Hall, University of California, San Francisco, San Francisco, CA, United States; ^8^Laboratory of Pathology, National Cancer Institute, National Institutes of Health (NIH), Bethesda, MD, United States; ^9^Department of Laboratory Medicine and Pathology, University of Minnesota Medical School, Minneapolis, MN, United States; ^10^Neurosurgery Unit for Pituitary and Inheritable Diseases, National Institute of Neurological Disorders and Stroke, Bethesda, MD, United States; ^11^Newborn Screening Program, Wadsworth Center, New York State Department of Health, Albany, NY, United States; ^12^Epidemiology Branch, Division of Intramural Population Health Research, Eunice Kennedy Shriver National Institute of Child Health and Human Development (NICHD), National Institutes of Health (NIH), Bethesda, MD, United States

**Keywords:** pituitary neuroendocrine tumor, Cushing's disease, DICER1, disease-modifying gene, corticotropinoma

## Abstract

**Context:** The DICER1 syndrome is a multiple neoplasia disorder caused by germline mutations in the *DICER1* gene. In DICER1 patients, aggressive congenital pituitary tumors lead to neonatal Cushing's disease (CD). The role of *DICER1* in other corticotropinomas, however, remains unknown.

**Objective:** To perform a comprehensive screening for *DICER1* variants in a large cohort of CD patients, and to analyze their possible contribution to the phenotype.

**Design, setting, patients, and interventions:** We included 192 CD cases: ten young-onset (age <30 years at diagnosis) patients were studied using a next generation sequencing panel, and 182 patients (170 pediatric and 12 adults) were screened via whole-exome sequencing. In seven cases, tumor samples were analyzed by Sanger sequencing.

**Results:** Rare germline *DICER1* variants were found in seven pediatric patients with no other known disease-associated germline defects or somatic *DICER1* second hits. By immunohistochemistry, DICER1 showed nuclear localization in 5/6 patients. Variant transmission from one of the parents was confirmed in 5/7 cases. One patient had a multinodular goiter; another had a family history of melanoma; no other patients had a history of neoplasms.

**Conclusions:** Our findings suggest that *DICER1* gene variants may contribute to the pathogenesis of non-syndromic corticotropinomas. Clarifying whether *DICER1* loss-of-function is disease-causative or a mere disease-modifier in this setting, requires further studies.

**Clinical trial registration:** ClinicalTrials.gov: NCT00001595.

## Introduction

Multiple molecular mechanisms causing pituitary neuroendocrine tumors (PitNETs) have been identified, but the genetic defects underlying such neoplasms remain unknown for the majority of the sporadic cases. The most common somatic genetic defects in somatotropinomas and corticotropinomas are activating somatic mutations in the *GNAS* (guanine nucleotide-activating alpha-subunit) and *USP8* (ubiquitin-specific protease 8) genes, respectively. On the other hand, 5–8% of PitNETs arise in a familial setting, either isolated (familial isolated pituitary adenoma) or as part of a syndrome affecting other organs, such as multiple endocrine neoplasia type 1 and type 4, McCune-Albright syndrome and Carney complex, and more rarely, within the DICER1 and succinate dehydrogenase-related syndromes (1).

The *DICER1* gene (14q32.13) encodes a ubiquitously expressed enzyme containing two endoribonuclease III domains (RNase IIIa and IIIb), required for the cleavage of precursor molecules into mature microRNAs (2–4). Germline or mosaic loss-of-function (LOF) *DICER1* mutations predispose to DICER1 syndrome or pleuropulmonary blastoma (PPB) familial tumor and dysplasia syndrome, an autosomal dominant condition causing PPB and multiple other tumors (5, 6). Pituitary blastoma (PitB) is a recently described component of this syndrome, causing severe neonatal Cushing's disease (CD) due to a malignant congenital pituitary tumor, with or without other associated tumors (7–9). Deleterious germline and somatic *DICER1* variants have also been reported in various sporadically occurring neoplasms (10–12).

The clinical presentation of the DICER1 syndrome is quite variable, and PitB displays low penetrance (8). Growing evidence has demonstrated the role of miRNAs and long non-coding RNAs in pituitary tumorigenesis (13, 14). Nevertheless, the specific role of *DICER1* in pituitary function and tumorigenesis remains unknown. Therefore, we have performed a thorough screening for *DICER1* variants in CD patients using targeted next generation sequencing (NGS) and whole-exome sequencing (WES). We describe, for the first time, rare variants in the *DICER1* gene in seven patients presenting with isolated corticotropinomas and CD arising during childhood.

## Subjects and Methods

### Patients and Samples

We studied 170 pediatric (≤18 years at disease onset) and 12 adult CD patients who are part of a large cohort recruited at the National Institutes of Health (NIH) Clinical Research Center between 1997 and 2018 under the research protocol 97-CH-0076 (ClinicalTrials.gov: NCT00001595). The *Eunice Kennedy Shriver* National Institute of Child Health and Human Development Institutional Review Board approved the study, and informed assent/consent was obtained from all the patients and their parents or guardians. Clinical data were obtained directly from the patients and/or from their medical records. For the patients and their parents, if available, DNA was extracted either from peripheral blood samples using the Maxwell 16 Blood DNA Purification Kit in a Maxwell 16 Instrument (Promega AS1015 and AS3050) or from saliva using the Oragene-Dx collection kit and the PrepIT-L2P DNA extraction kit (DNA Genotek OGD-500 and PT-L2P-45), according to the manufacturer's protocols. Additional studies in this cohort have been reported elsewhere (15–22).

We also studied ten young patients (age <30 years at diagnosis) with diagnosed familial or sporadic CD; these cases are part of a multicenter study performed at the Biocruces Bizkaia Health Research Institute and the Department of Endocrinology and Pediatric Endocrinology at the Cruces University Hospital (Barakaldo, Spain). Clinical data were provided by the clinicians responsible for these patients and written informed consent was obtained from all participants or their parents. The study was approved by the local Ethics committees. Extraction and purification of DNA from peripheral blood leukocytes was performed using the MagPurix Blood DNA Extraction Kit 200, according to the manufacturer's instructions (Zinexts Life Science Corp., Taiwan).

When possible, unstained tumor slides were obtained from the corresponding departments of Anatomical Pathology. After manual delimitation of the tumor area, DNA was extracted from unstained sections using the Pinpoint Slide DNA Isolation System (Zymo Research D3001).

Written, informed consent was obtained from the individuals and/or minors' legal guardian for the publication of any potentially identifiable images or data included in this article.

### Design of a Targeted Gene Panel and Screening via Next-Generation Sequencing

A targeted panel for PitNETs was designed to include nine genes (*AIP, CDKN1B, DICER1, GNAS, MEN1, PRKAR1A, SDHB, SDHC*, and *SDHD*) associated with human pituitary tumors in online databases, including PubMed (https://www.ncbi.nlm.nih.gov/pubmed/), Human Gene Mutation Database (HGMD, https://portal.biobase-international.com) and Online Mendelian Inheritance in Man (https://www.ncbi.nlm.nih.gov/omim). The design of the panel was performed by Ion AmpliSeq™ Designer (Thermo Fisher Scientific, USA) and oligonucleotides against exons, flanking intronic and untranslated (UTR) regions were included. Library preparation was done using the Ion Ampliseq Library Kit v2.0 (Thermo Fisher Scientific) according to manufacturer's instructions. Samples were then sequenced using the Personal Genome Machine system with an Ion 316™ Chip (Thermo Fisher Scientific). Base calling, read filtering, alignment to the reference human genome GRCh37/hg19, and variant calling were done using Ion Torrent Suites (Thermo Fisher Scientific). Further QC analysis, coverage analysis, and variant filtering were completed with the Ion Reporter Software (Thermo Fisher Scientific). Coverage depth and read quality were also evaluated with the Integrative Genomics Viewer (broadinstitute.org).

Germline DNA samples from the 182 patients studied at NIH were submitted for WES at the University of Minnesota Genomics Center (UMGC, 91 patients) or Novogene (91 patients) and analyzed at UMGC. Targeted capture libraries were generated using the Agilent QXT v5 + UTRs (UMGC) or SureSelect Human All Exon v6 (Novogene) kits. Samples were sequenced on an Illumina HiSeq 2000 platform producing 100 base pair paired-end reads (UMGC) or on an Illumina HiSeq 2500 platform producing 150 bp paired-end reads (Novogene), while tumor samples were sequenced on a HiSeq 2500 platform producing 1,265 bp paired-end reads. WES results for all samples were analyzed together. FASTQ files were processed using a Genome Analysis Toolkit (GATK) v3.7 based pipeline, including BWA-MEM v0.7.17 for alternate contig aware alignment to the hg38 reference genome (GRCh38_full_analysis_set_plus_decoy_hla.fa), Picard Tools v2.6.0 to mark duplicates (picard, retrieved from http://broadinstitute.github.io/picard/), and GATK for indel realignment, base quality recalibration, genotyping (HaplotypeCaller), variant quality score recalibration, and to split multiallelic sites (23, 24). ANNOVAR was used to determine the effect of coding variants using both the RefSeq and UCSC gene sets, including putative amino acid changes, distance to intron-exon boundary, the creation or removal of a stop-codon, and location within known non-coding RNAs (25). Non-synonymous variants were annotated based on their computationally predicted deleteriousness using information from dbNSFP. All variants were annotated for their presence and frequency in multiple variant collections (e.g., dbSNP, 1,000 Genomes, and internal WES datasets totaling over 10,000 samples). All the results were filtered to include only variants with a Phred-like score ≥30. The median number of on-target reads generated per sample was 451 million, resulting in a median target coverage of 60X (89% of targets covered at >20X). For *DICER1*, the first three exons (of the 29 total exons) represent 5′ untranslated region (5′ UTR) and had a median percent covered at 20X of 0.0%. Of the 26 coding exons in DICER1, 20 had a median percent covered at 20X of 95% or greater. Exons 7 (73.1%), 8 (41.9%), 9 (20.2%), 10 (90.0%), 28 (46.7%), and 29 (80.6%) each had a median percent covered at 20X of <95%.

### *In silico* Analyses and Variant Classification

The impact of non-synonymous variants on protein structure and function was assessed *in silico* using Mutation Taster (http://www.mutationtaster.org/), MutPred (http://mutpred.mutdb.org/), Polyphen 2 (http://genetics.bwh.harvard.edu/pph2/), PROVEAN (Protein Variation Effect Analyzer) (http://provean.jcvi.org/index.php), SIFT (Sorting Intolerant from Tolerant, http://sift.jcvi.org/), SNPs and Go (http://snps.biofold.org/snps-and-go/snps-and-go.html), and VarSome (https://varsome.com). Variants were classified following the standards and guidelines from the American College of Medical Genetics and Genomics (26). We focused our study on coding variants with a minor allele frequency (MAF) <1% in gnomAD (http://gnomad.broadinstitute.org).

### Sanger Sequencing

Variants of interest were confirmed by bidirectional direct sequencing using the BigDye Terminator 3.1 Cycle Sequencing Kit (Thermo Fisher Scientific 4337456) in a 3500xL Genetic Analyzer (Applied Biosystems, primer sequences, and conditions are available from the authors, on request). All variants were annotated according to the GenBank reference sequences NM_030621.4 and NP_085124.2. When available, DNA samples from the patients' parents were screened likewise. Since all the selected variants were heterozygous at the germline level, loss of heterozygosity (LOH) in the tumors was also investigated. In addition, the sequence encoding the RNAse IIIb domain (amino acids 1666–1824) of DICER1 was sequenced from tumor DNA samples. Previously reported clinical associations were searched in the databases ClinVar (https://www.ncbi.nlm.nih.gov/clinvar), catalog of Somatic Mutations in Cancer (COSMIC, https://cancer.sanger.ac.uk/cosmic), and HGMD. In selected cases, the primers 5′-CTTCCACCCCTCCAACTCAT-3′ and 5′-TGGAGTTACTGTTGGCTTCCT-3′ were used to amplify and sequence a region of 146 bp covering the *USP8* mutational hotspot in tumor DNA.

### Immunohistochemistry

Immunohistochemical staining for DICER1 was performed using 1:200 polyclonal rabbit anti-DICER1 primary antibody (Sigma-Aldrich HPA000694), and amplification with 1:1,000 Biotin-SP AffiniPure Goat Anti-rabbit IgG and 1:500 peroxidase streptavidin (Jackson ImmunoResearch Laboratories 111-065-144 and 016-030-084, respectively). Samples were developed using ImmPACT DAB peroxidase (HRP) substrate (Vector SK-4105). The rest of the protocol has been detailed elsewhere (19). Images were acquired using a Keyence BZ-X710 microscope and processed with the BZ-X Analyzer software (Keyence).

### Statistical Analyses

The Prism v8.2.1 software (GraphPad Software) was used for all statistical analyses. Parametric data are presented as mean ± standard deviation and non-parametric data are presented as median and interquartile range. Gene variant frequencies in the study population were compared with the frequencies reported in public databases using the chi-squared or the Fisher's exact test, as appropriate. Results were considered statistically significant when *P* < 0.05.

## Results

### Study Population

The Biocruces Bizkaia Health Research Institute included 10 probands (60% females), with mean age at diagnosis of 17.9 (7.5–29.0) years and median tumor size of 17.4 (9–20) mm. The NICHD cohort included 182 patients, 56.6% (*n* = 103) females and 43.4% (*n* = 79) males. The median age at first symptoms and diagnosis were 10 (8–13) and 12.8 (10.5–15.5) years among the pediatric patients, while adult patients had a mean age of 38.8 ± 9.5 at first symptoms and 42 ± 12.6 at diagnosis. The median maximum tumor diameter was 5.8 (4–8) mm; 84.1% (*n* = 153) of patients had a microadenoma, 12.1% (*n* = 22) had a macroadenoma, 2.7% (*n* = 5) had multiple adenomas, and two patients had no tumor identifiable by histopathology (CD was proven in these cases by means of bilateral inferior petrosal sinus sampling, BIPSS, and resolution of hypercortisolemia after surgery). Fifteen patients had familial presentation and the rest were apparently sporadic. Except for somatic genomic instability in Case 2 (detailed below), none of the patients with *DICER1* variants reported here have other known germline PitNET-associated genetic defects.

### Patients With *DICER1* Gene Variants of Interest

**Figure 1:**
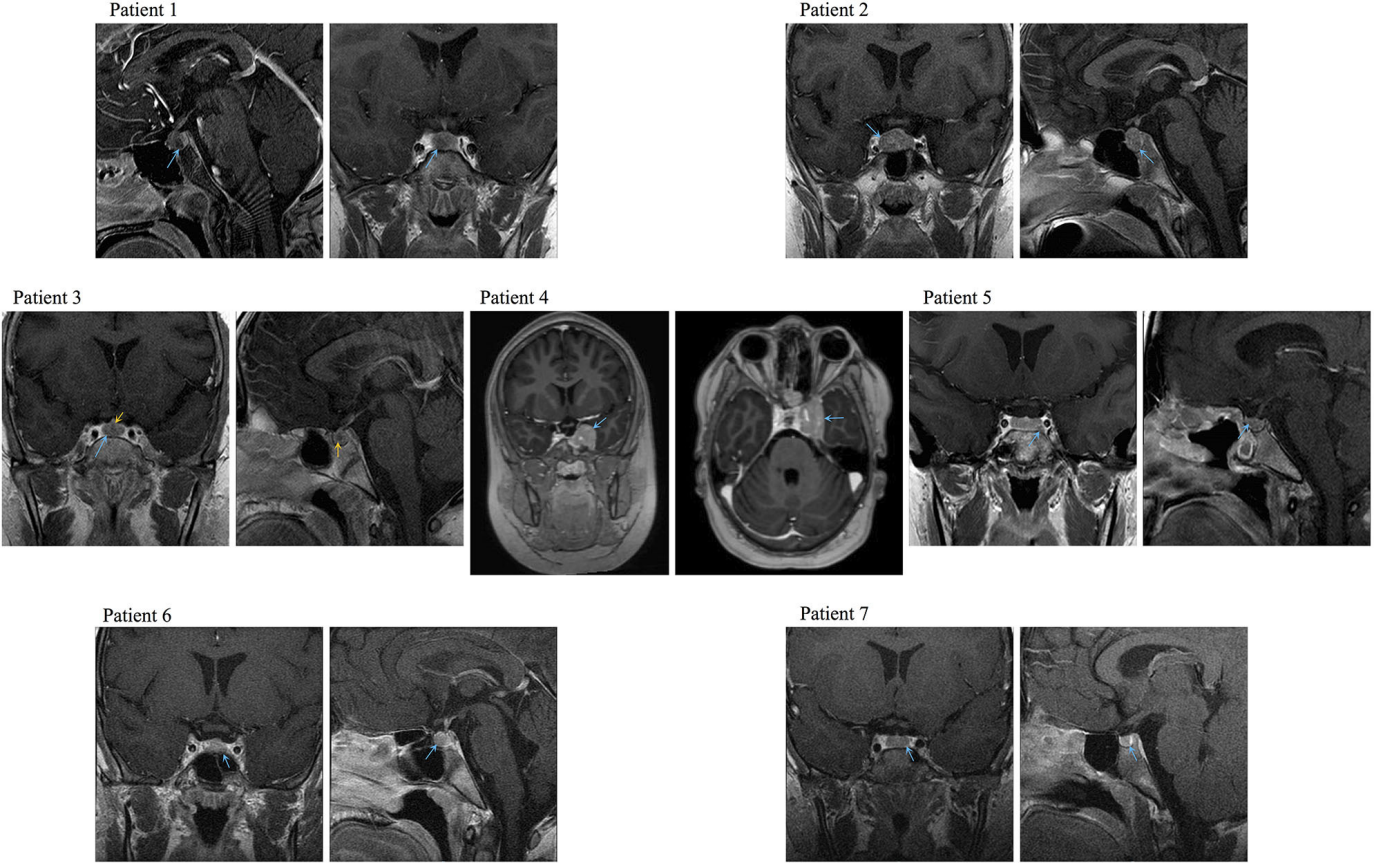
Representative preoperative MRI images of the PitNETs diagnosed in Cases 1–4, 6, and 7. Image for Case 5 was obtained after her first surgery (preoperative image not available). Blue arrows: tumors, yellow arrows: medial lobe cyst.

**Figure 2:**
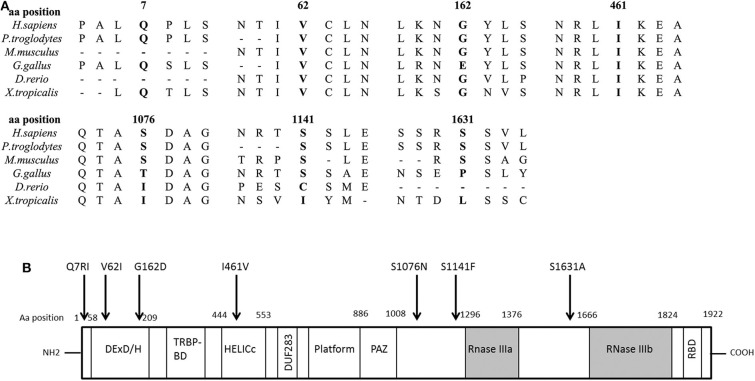
DICER1 protein comparison against different species and structure diagram of the protein. **(A)** Alignment of the DICER1 protein sequence across species. Position of the identified amino acid variants is presented in bold characters. Most of the identified variants are well conserved across species. **(B)** Scheme of the structure of the DICER1 protein and localization of studied variants. The main functional domains of DICER1 are the helicase domain including DEXD/H (DEAD box), TRBP-BD (trans-activation response RNA-binding protein-binding), HELICc (helicase conserved carboxy-terminal domain), and DUF283 (domain of unknown function), followed by a platform domain which separates the PAZ (Piwi/Argonaute, Zwille) domain from the two distinct RNase III domains (RNase IIIa and RNase IIIb) and the double-stranded RNA-binding domain (RBD). Localization of the *DICER1* variants of interest described in this study is indicated by an arrow. Aa, amino acid.

We found seven germline heterozygous missense *DICER1* gene variants of interest each in an unrelated patient presenting with non-syndromic, apparently sporadic CD during childhood (one patient from the Biocruces Bizkaia cohort and six from the NICHD cohort). The patients' clinical, biochemical, and histopathological data are summarized in Table 1, and representative MRI images are presented in Figure 1. The genetic characterization and previous reports of the variants are detailed in Table 2 and Figure 2. In addition to these infrequent variants, we found that other, more common *DICER1* variants, were overrepresented in our cohort, compared with the general population, as presented in Supplemental Table 1.

**Table 1:** Clinical and genetic characterization of patients harboring *DICER1*variants of interest.

**Case**	**Gender, race (origin)**	**Age at diagnosis (years)**	**Clinical presentation**	**Biochemistry**	**Histopathology**	**Tumor size**	**Treatment**	**Current status**	***DICER1* gene variant (inheritance)**	**Genetic findings in tumor**
1	M, White (USA)	7.8	Weight gain (weight + 6.9 SDS), growth rate deceleration (height + 0.8 SDS), acanthosis nigricans, fatigue, mood disturbances	UFC: 13.6 μg/24 h (6.8 × ULN); midnight serum cortisol: 10.2 μg/dl; CRH stimulation test: 151% increase in cortisol and 512% increase in ACTH; BIPSS: maximum central-peripheral ratio of 15.5, left-right ratio of 11.6	PitNET (no IHC)	Microadenoma (7 × 6 × 3 mm)	TSS × 2	Remission	c.20A>G, p.Q7R heterozygous (maternal origin)	c.20A>G, p.Q7R heterozygous (no LOH); RNase IIIb domain wt; *USP8* hotspot wt
2	F, White (Brazil)	8.5	Weight gain (weight + 2.8 SDS), growth rate deceleration (height −1 SDS), hirsutism, and mood disturbances	UFC: 191.4 μg/24 h (9.6 × ULN); midnight serum cortisol: 17.5 μg/dl; CRHST: 175.6% increase in cortisol and 500.7% increase in ACTH	Corticotropinoma	Macroadenoma (12 × 18 mm) with invasion to the medial wall of the right cavernous sinus	TSS	Remission	c.184G>A, p.V62I heterozygous (unknown origin: mother wt, father n/a)	c.184G>A, p.V62I heterozygous (no LOH); RNase IIIb domain wt; *USP8* hotspot wt; chromosomal instability by CNV analysis of WES data
3	M, African American (USA)	12.8	Weight gain (weight + 3.2 SDS), headaches, tinea corporis, striae, facial plethora, fatigue, dorsocervical fat pad, hypertension, and growth rate deceleration (height −1.9 SDS)	UFC: 209 μg/24 h (5.6 × ULN); midnight serum cortisol: 14.9 μg/dl; 08:00 a.m. serum cortisol post-overnight LDDST: 2 μg/dl; CRHST: 135% increase in cortisol and 727% increase in ACTH	Corticotropinoma	Microadenoma (4 × 3 × 2 mm)	TSS	Remission	c.485G>A, p.G162D heterozygous (unknown origin: mother wt, father n/a)	485G>A, p.G162D heterozygous (no LOH); RNase IIIb domain wt; *USP8* hotspot wt
4	F, White (Spain)	9	Visual impairment, striae, proximal myopathy, hyperandrogenism and central obesity (BMI +3.4 SD)	UFC: 914.1 μg/24 h (6.8 × ULN); IGF-1: 718 ng/ml	Corticotropinoma (negative IHC for prolactin, GH, FSH, LH, and TSH)	Macroadenoma (25 × 10 × 17 mm)	TSS x2, ketoconazole, cabergoline, radiotherapy	Remission	c.1381A>G, p.I461V heterozygous (paternal origin)	c.1381A>G, p.I461V heterozygous (no LOH); RNase IIIb domain wt; *USP8* hotspot wt
5	F, Asian (China/USA)	16.3	CD-related: weight gain (weight +1.8 SDS), growth rate deceleration (height−0.6 SDS), acne, acanthosis, nigricans, facial plethora, striae, dorsocervical fat pad, hypertension, secondary amenorrhea, and mood disturbances. Other: nighttime hypoxia, polycythemia, multinodular goiter	UFC: 87.2 μg/24 h (1.6 × ULN); midnight serum cortisol: 4.7 μg/dl; CRHST: 145% increase in cortisol and 1,025% increase in ACTH*	Corticotropinoma (Crooke's cell adenoma)	Microadenoma (5 mm) with invasion to the left cavernous sinus	TSS × 3, gamma knife, ketoconazole	Active	c.3227G>A, p.S1076N heterozygous (paternal origin)	c.3227G>A, p.S1076N heterozygous (no LOH). RNase IIIb domain wt, *USP8* hotspot wt
6	M, White (USA)	10.8	Weight gain (weight 4.5 SDS), growth rate deceleration (height −0.6 SDS), facial plethora, fatigue and mood disturbances, dorsocervical fat pad, supraclavicular fat deposition, proximal muscle weakness, and hypertension	UFC: 389 μg/24 h (10.5 × ULN), midnight serum cortisol: 15.2 μg/dl; midnight salivary cortisol: 769 ng/dl (expected <100); 08:00 a.m. serum cortisol post-overnight LDDST: 15.4 μg/dl; CRHST: increase 61% in cortisol and 238% in ACTH	Corticotropinoma (Crooke's cell adenoma)	Microadenoma (7 mm)	TSS	Remission	c.3422C>T, p.S1141F heterozygous (maternal origin)	c.3422C>T, p.S1141F heterozygous (no LOH). RNase IIIb domain wt, *USP8* hotspot wt
7	F, White (USA)	8.4	Headache, dizziness, weight gain (weight 4.4 SDS) and growth rate deceleration (height −0.4 SDS)	UFC: 141 μg/24 h (7.1 × ULN); midnight serum cortisol: 11.5 μg/dl; 08:00 a.m. serum post-overnight LDDST: 9.7 μg/dl; CRHST: 90% increase in cortisol and 306% increase in ACTH; BIPSS: maximum central-peripheral ration of 70.4; left-right ratio of 9.8	Corticotropinoma	Microadenoma (5 mm) with invasion of the medial wall of the left cavernous sinus	TSS	Remission	c.4891T>G, p.S1631A heterozygous (paternal origin)	c.4891T>G, p.S1631A heterozygous (no LOH). RNase IIIb domain wt, *USP8* hotspot wt

*BIPSS, bilateral inferior petrosal sinus sampling; CRHST, CRH-stimulation test; IHC, immunohistochemistry; LDDST, low-dose dexamethasone suppression test; LOH, loss of heterozygosity; n/a, not available; PitNET, pituitary neuroendocrine tumor; SDS, standard deviation score; TSS, transsphenoidal surgery; UFC: urinary free cortisol; ULN, upper limit of normal; wt, wild type*.

* *Results obtained before second surgery*.

**Table 2:** *DICER1* variants of interest identified among pediatric patients with pituitary neuroendocrine tumors.

**Case**	**HGVS nomenclature: DNA, protein**	**Location, variant type**	**dbSNP ID**	**MAF in gnomAD (v2.1.1)**			***In silico*** **prediction**							**Pathogenic associations**
				**Exomes**	**Genomes**	**Exomes and genomes**	**Mutation taster**	**MutPred 2**	**PolyPhen 2**	**Provean**	**SIFT**	**SNPs and GO**	**Varsome**	
1	c.20A>G, p.Q7R	Exon 4, missense	rs117358479	0.1693	0.1720	0.1696	P	n/a	B	N	D	N	Likely benign	ClinVar RCV000493327.2: hereditary cancer-predisposing syndrome, likely benign (27, 28). RCV000206043.5: DICER1 syndrome, benign. RCV000120631.2, RCV000433632.1: not specified, likely benign. RCV000344796: PPB, likely benign. COSMIC: found in one case of breast cancer (29)
2	c.184G>A, p.V62I	Exon 5, missense	rs746671039	0.0008	n/a	0.0008	DC	Altered metal binding	B	N	D	N	Likely benign	HGMD CM1614023: RMS (two cases), germline, disease-causing (30, 31). ClinVar RCV000654380.1: DICER1 syndrome, VUS (32). COSMIC: found in one case of colon adenocarcinoma, confirmed somatic (33)
3	c.485G>A, p.G162D	Exon 7, missense	rs142815547	0.0586	0.1848	0.0726	DC	NP	B	N	T	DC	Likely benign	ClinVar: RCV000234323.4: DICER1 syndrome, benign. RCV000605038.1: not specified, benign. RCV000494493.1: hereditary cancer-predisposing syndrome, likely benign
4	c.1381A>G, p.I461V	Exon 11, missense	rs141163928	0.0179	0.0032	0.0163	DC	NP	B	N	T	N	Likely benign	ClinVar: RCV000229670.4: DICER1-related PPB cancer predisposition syndrome, VUS. RCV000566185.1: hereditary cancer-predisposing syndrome, VUS. RCV000331637.1: PPB, likely benign
5	c.3227G>A, p.S1076N	Exon 22, missense	rs778494781	0.0036	0.0032	0.0035	P	NP	B	N	T	N	Likely benign	ClinVar: RCV000476968.3: DICER1 syndrome, VUS
6	c.3422C>T, p.S1141F	Exon 23, missense	rs780815020	0.0016	0.0032	0.0018	DC	NP	B	N	D	N	Likely benign	ClinVar: RCV000477099.5: DICER1 syndrome, VUS. RCV000570654.1: hereditary cancer-predisposing syndrome, VUS. COSMIC: Found in one case of malignant melanoma, confirmed somatic (34)
7	c.4891T>G, p.S1631A	Exon 25, missense	rs145551486	0.1651	0.1114	0.1591	P	NP	B	N	T	N	Likely benign	ClinVar RCV000205231.5: DICER1 syndrome, likely benign. RCV000567615.1: hereditary cancer-predisposing syndrome, likely benign. RCV000611518.1: not specified, likely benign. RCV000301109.1: PPB, likely benign

*B, benign; D, damaging; DC, disease causing; Del, deleterious; MAF, minor allele frequency; N, neutral; n/a, not available; NP, not pathogenic; P, polymorphism; PD, possibly damaging; PPB, pleuropulmonary blastoma; RMS, rhabdomyosarcoma; T, tolerated; VUS, variant of uncertain significance*.

*All variants were annotated according to NCBI GenBank reference sequences NM_030621.4 and NP_085124.2*.

#### Case 1

After a 1-year history of weight gain, reduced growth velocity, acanthosis nigricans, fatigue, and mood disturbances, this boy was diagnosed with CD at age 7.8 years. He had an elevated midnight serum cortisol, a positive CRH stimulation test (CRHST), and a possible pituitary microadenoma by magnetic resonance imaging (MRI); CD was confirmed by BIPSS. Remission was only achieved after two transsphenoidal surgeries and, in addition to a histologically confirmed PitNET, an intermediate lobe cyst was also resected. His family history is relevant for the diagnosis of skin melanoma in his mother, maternal grandfather, and a maternal great-uncle. The variant c.20A>G, p.Q7R (previously reported as a likely benign change in DICER1 syndrome patients and as a somatic change in a case of breast cancer) was detected in the patient, as well as in his mother and maternal grandfather.

#### Case 2

This female patient was diagnosed with CD at age 8.6 years, after having developed excessive weight gain, reduced growth velocity, hirsutism, and mood disturbances over the previous 1.5 years. She had elevated urinary free cortisol (UFC) and midnight serum cortisol, and her pituitary MRI revealed a macroadenoma. A corticotropinoma with invasion of the medial wall of the right cavernous sinus was resected, and the patient achieved remission. The variant identified in this case (c.184G>A, p.V62I), is extremely rare in the general population. It has been reported before as a germline variant probably associated with two cases of rhabdomyosarcoma, as a probable variant of uncertain significance (VUS) in DICER1 syndrome, and as a somatic change in colon adenocarcinoma.

#### Case 3

At age 9 years, this male patient developed excessive weight gain, headaches, tinea corporis, red striae, facial plethora, and fatigability. During his clinical evaluation, a dorsocervical fat pad, hypertension, and reduced growth velocity were also noted. He was diagnosed with CD at age 12.8 years, having elevated UFC and midnight serum cortisol, as well as a positive overnight low-dose dexamethasone suppression test (LDDST) and an MRI showing a microadenoma. The patient developed hypocortisolism after transsphenoidal surgery, and a corticotropinoma was histologically confirmed. He carried the variant c.485G>A, p.G162D, which has been reported before in patients with DICER1 syndrome as a benign or likely benign variant, and as a somatic change in a patient with Wilms tumor.

#### Case 4

At age 9 years, this female patient developed visual impairment, striae, proximal myopathy and clinical features of hyperandrogenism. Blood tests detected increased UFC and IGF-1, and the patient was diagnosed with CD. MRI showed a pituitary macroadenoma and transsphenoidal surgery was performed. Tissue immunohistochemistry was positive for ACTH. After surgery, the patient required treatment with ketoconazole and cabergoline, and 1 year later, a second surgery was performed due to persistent cortisol hypersecretion. At age 11 years, the patient presented with a second recurrence, which was successfully treated by radiotherapy. IGF-1 levels were within the normal range after the second surgery (127 ng/ml). At age 13 years, she presented with panhypopituitarism, obesity, and insulin resistance. The variant c.1381A>G, p.I461V in exon 11 of *DICER1* was found in the patient and in her apparently healthy father.

#### Case 5

Around age 13 years, this female patient developed excessive weight gain with central fat distribution, reduced growth velocity, acne, acanthosis nigricans, facial plethora, striae, dorsocervical fat pad, hypertension, secondary amenorrhea, and mood disturbances. She had elevated UFC and midnight serum cortisol, and an MRI showing a microadenoma; a diagnosis of CD was established at age 16.1 years. During her first transsphenoidal surgery, invasion of the wall of the cavernous sinus was documented and histopathological examination confirmed a Crooke's cell corticotropinoma. A second transsphenoidal surgery resulted in remission; unfortunately, she presented with a new recurrence one year later and radiotherapy was then administered, followed by treatment with ketoconazole. The patient has a personal history of nighttime hypoxia, polycythemia, and multinodular goiter, but no family history of neoplasms. She carried the *DICER1* variant c.3227G>A, p.S1076N, reported in ClinVar as a VUS for DICER1 syndrome, which she inherited from her apparently healthy father.

#### Case 6

At age 9 years, this previously healthy boy developed excessive weight gain with central fat distribution, growth rate deceleration, facial plethora, fatigability, and mood disturbances. During clinical examination, dorsocervical fat pad, supraclavicular fat deposition, proximal muscle weakness, and hypertension were also found. He was diagnosed with CD at age 10.8 years. Relevant findings during his diagnostic workup included combined dyslipidemia, as well as elevated UFC, unsuppressed midnight serum and salivary cortisol, positive LDDST, and CRHST and a pituitary microadenoma by MRI. Remission was achieved after transsphenoidal surgery and histopathological examination revealed a Crooke's cell corticotropinoma. The variant c.3422C>T, p.S1141F, detected in the patient and his mother, has been classified as a VUS in patients with DICER1 syndrome and has also been reported as a somatic change in one case of malignant melanoma.

#### Case 7

This female patient had a history of episodes of headache and dizziness starting at age 3 years. At age 5 she also developed, excessive weight gain with central fat distribution and growth rate deceleration. At age 8.4 the patient was diagnosed with CD, following biochemical tests showing elevated UFC and midnight serum cortisol and an MRI demonstrating a pituitary microadenoma. A corticotropinoma with invasion of the medial wall of the left cavernous sinus was resected, leading to disease remission. The patient carried the variant c.4891T>C, p.S1631A; this change was inherited from her father, who is affected with hypertension and hyperuricemia, but has no history of neoplasms. This variant has been reported as a likely benign change in patients with DICER1 syndrome and other associated phenotypes.

### Genetic Screening and Immunohistochemistry in Corticotropinoma Tissues

At the somatic level, none of the cases presented LOH for the *DICER1* germline variants of interest, and no additional variants were found in the gene region encoding the RNase IIIb domain (end of exon 25–28). None of these seven patients carried somatic hotspot *USP8* mutations either. Interestingly, the corticotropinoma found in case 2 (previously published as PT282.03) displays somatic chromosomal instability, detected by WES copy number variation analysis, as we have recently reported (22).

**Figure 3:**
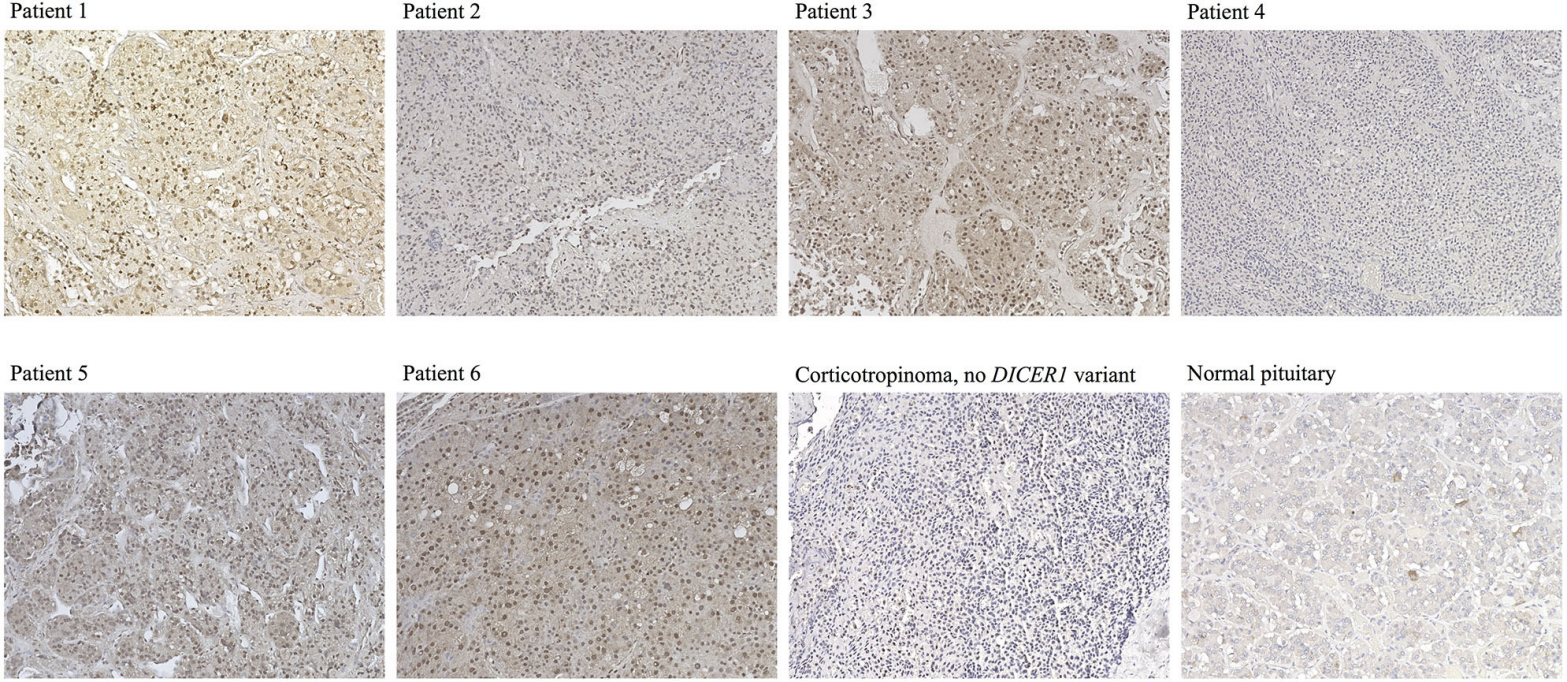
Immunostaining for DICER1 in PitNETs and normal pituitary. Representative images are presented for Cases 1–6 (no slides were available for staining for Case 7), as well as for a corticotropinoma from a patient with no *DICER1* variants of interest and for a normal pituitary specimen. Magnification for all images: 20 ×.

DICER1 immunohistochemistry of the corticotropinomas (Figure 3), revealed predominantly nuclear but also cytoplasmic staining in most of the cells in Patients 1–3 and 5–6, in contrast with the purely cytoplasmic expression detected in PitNETs with no *DICER1* variants of interest and in the normal pituitary gland. In contrast, tumor tissue from Patient 4 presented exclusively cytoplasmic staining.

## Discussion

We hypothesized that *DICER1* variants could play a role in the development and function of corticotroph cells, as well as in corticotroph tumorigenesis, based on the association of *DICER1* LOF with congenital CD due to PitB. Supporting this hypothesis, we detected seven infrequent missense germline *DICER1* variants in an equal number of pediatric patients with apparently sporadic corticotropinomas. In addition, we found an overrepresentation of other more common *DICER1* variants in our cohort, compared with the general population. The study of a unique large cohort of young CD patients has allowed us to identify such rare genetic alterations, which might very infrequent in the general population of patients with PitNETs. We confirmed variant transmission from one of the parents in five of the cases of interest, but except for multinodular goiter in Case 5, no familial or personal history of any of the components of the DICER1 syndrome was found. There was, however, a history of familial melanoma in *DICER1* variant carrier relatives of Case 1, and although not considered to be associated to the syndrome, somatic *DICER1* defects have been described in melanoma cases. In the two cases where variant inheritance was not proven, only one of the parents was available for testing; thus, we could not determine whether those variants appeared *de novo*.

To date, the only pituitary lesion clearly associated with *DICER1* mutations is PitB, an infrequent undifferentiated and aggressive subtype of PitNET first described by Scheithauer et al. (7, 35). Two main studies support this genetic association: a series of 13 cases which confirmed *DICER1* mutations in 11 patients (nine proven germline), and a report of an infant with a germline frameshift deletion and a somatic missense variant causing aggressive CD (8, 9). In a recent report, a *DICER1* mutation carrier was diagnosed with a microprolactinoma and papillary thyroid cancer with multinodular goiter; she had a daughter who died of ovarian cancer (36). Although the occurrence of a PitNET in this patient could be a coincidence, an association with the *DICER1* defect cannot be ruled out.

All the variants identified in our patients have been classified previously as benign, likely benign, or VUS regarding their association with the DICER1 syndrome and other reported phenotypes, but no experimental data were found in the literature to validate their functional effects. They could potentially affect the tumor suppressor function of DICER1, although they are not clustered together in a specific protein domain (Figure 2). Performing the functional experiments required to analyze the impact of these variants was, however, beyond the scope of this manuscript. Causality has not been proven between *DICER1* loss-of-function and PitNETs other than PitB, and our patients present a phenotype quite distinct from the DICER1 syndrome. Therefore, although non-syndromic corticotropinomas could be a new *DICER1-*associated phenotype, at the moment we cannot confidently determine whether the variants found in our patients are indeed causative or if they could act as disease modifiers.

*DICER1* variants causing the DICER1 syndrome usually result in truncated proteins and are most often inherited, but appear *de novo* in 10–20% of cases (37). Although the majority of patients carry germline heterozygous variants, 10% of cases are due to mosaic defects, and most patients harbor second hits (somatic variants or, less frequently, LOH) (38). While germline variants can affect any region of the protein, reported somatic variants are localized in hotspots within the RNase IIIb domain and lead to reduced processing of mature 5′ miRNA strands, shifting the mature miRNA expression toward 3′-derived miRNAs, a potential oncogenic mechanism (39). In contrast, the variants found in our patients are non-truncating and lie outside the RNase IIIb domain, and no second hits were identified. Such findings were not completely unexpected, given the relatively benign phenotype of the tumors (none of the patients presented with pituitary blastoma or neonatal disease), and could indicate the presence of alternative causative genetic defects, meaning that *DICER1* variants could be merely contributory in this setting. Interestingly, none of these CD cases harbored somatic *USP8* mutations, which are rather frequent among sporadic CD cases, including in our cohort (17).

Another important observation from our study is the preferential nuclear localization of DICER1 by immunohistochemistry. Under physiological conditions, most of the functions of the DICER1 protein take place in the cytoplasm, but a small amount of the protein localizes to the nucleus, where it takes part in non-canonical RNAi processing, and DNA damage triggers its nuclear accumulation (40, 41). Therefore, the staining pattern observed could indicate either a response to previously induced DNA damage or an abnormal subcellular localization of defective DICER1 proteins. Unfortunately, inconsistencies in antibody performance for DICER1 immunohistochemistry have been reported in the literature (42). Analyzing a larger collection of tumors would be required to determine whether such findings are either indicative of *DICER1* alterations, or a display of a physiological cellular response to tumorigenic stimuli. Of note, two patients had a histopathological diagnosis of Crooke's cell adenoma. Little is known about the molecular mechanisms underlying this relatively rare and particularly aggressive subtype of corticotropinoma, which represents 5.9% of the cases (*n* = 10) in our pediatric cohort (43). Whether *DICER1* defects could be linked to this phenotype requires further investigations.

In conclusion, the identification of seven pediatric patients with corticotropinomas harboring heterozygous *DICER1* variants revealed a possible role for *DICER1* gene defects in corticotroph tumorigenesis. Further studies are required to assess the functional effect of these variants on miRNA processing, as well as to explain the specific role of *DICER1* in the normal pituitary gland and in corticotroph tumorigenesis.

## Data Availability Statement

All datasets presented in this study are included in the article/Supplementary Material.

## Ethics Statement

Approval for this study was obtained from The Eunice Kennedy Shriver National Institute of Child Health and Human Development Institutional Review Board (research protocol 97-CH-0076 ClinicalTrials.gov: NCT00001595), and from Basque Clinical Research Ethics Committee (CEIC-E). Written informed assent or consent to participate in this study was provided by all the participants or legal guardians, as appropriate.

## Author Contributions

IM, LH-R, LC, and CS contributed in the conception and study design, data were collected by IM, LH-R, NPo, AG-G, IU, RM-S, AG-C, AA, IR, SG, FF, MK, ML, MQ, NPa, PC, JL, DK, and JM. Data analysis and interpretation was carried out by IM, LH-R, IR, SG, JM, LC, and CS. IM and LH-R prepared the manuscript. All authors contributed to manuscript revision and approval. All authors contributed to the article and approved the submitted version.

## Conflict of Interest

The authors declare that the research was conducted in the absence of any commercial or financial relationships that could be construed as a potential conflict of interest.
