# Supplementary material for: Rare Germline DICER1 Variants in Pediatric Patients With Cushing's Disease: What Is Their Role?
Source: Front Endocrinol (Lausanne). 2020 Jul 3;11:433. doi: 10.3389/fendo.2020.00433 (PMC7351020; doi:10.3389/fendo.2020.00433)
Supplement: Supplementary file 1 [file Table_1.DOCX]

Supplementary Material

**Supplementary table 1.** Additional *DICER1* variants found in the study cohorts.

| **HGVS nomenclature:**  **DNA, protein** | **Location in gene** | **Variant type** | **dbSNP ID** | **MAF in our cohort (%)** | **MAF in gnomAD v2.1.1 (%), *P* value compared with our cohort** | | |
| --- | --- | --- | --- | --- | --- | --- | --- |
|  |  |  |  |  | **gNOMAD exomes** | **gNOMAD genomes** | **gNOMAD exomes and genomes** |
| c.-146-83A>G | Intron 2 | Intronic | rs17091845 | 0.2604 | n/a | 3.549, ***P*<0.001** | |
| c.-46+2508T>G | Intron 3 | Intronic | Not in dbSNP | 0.2747 | n/a | | |
| c.-46+2609A>T |  |  | rs553196891 | 0.2747 | n/a | 0.0159, *P*=0.0669 | |
| c.-46+2751C>T |  |  | rs140969547 | 0.5495 | 0.9026, *P*=0.7687 | 1.4305, *P*=0.2570 | 1.3609, *P*=0.2512 |
| c.-46+2814G>A |  |  | rs58532636 | 0.8242 | n/a | 1.7171, *P*=0.3022 | |
| c.-20A>G | Exon 4 (5'UTR) |  | rs370105837 | 0.2747 | 0.0147, *P*=0.0536 | 0.0255, *P*=0.0985 | 0.0160, *P*=0.0579 |
| c.307+81A>C | Intron 5 |  | rs114886684 | 1.3736 | n/a | 1.4684, *P*=0.8811 | |
| c.307+200T>C |  |  | rs140623483 | 0.2604 | n/a | 0.5637, *P*=0.729 | |
| c.439-90T>G | Intron 6 |  | rs117349874 | 0.5495 | n/a | 0.3533, *P*=0.3720 | |
| c.573+116C>T | Intron 7 |  | rs1953535 | 3.2967 | n/a | 11.0190**, *P*<0.0001** | |
| c.735-75delT | Intron 8 |  | rs1394414999 | 3.5714 | n/a | 6.4531*, ***P*=0.0289** | |
| c.735-75dupT |  |  | rs75156371 | 0.8242 | n/a | 10.8924*, ***P*<0.0001** | |
| c.735-74G>T |  |  | rs79285101 | 2.1978 | n/a | 16.6133*, ***P*<0.0001** | |
| c.735-73C>T |  |  | rs556908107 | 0.2747 | n/a | 0.9679*, *P*=0.2522 | |
| c.904-65C>T | Intron 9 |  | rs67737119 | 9.0659 | n/a | 9.7119, *P*=0.6789 | |
| c.1377-4T>G | Intron 10 |  | rs192490028 | 0.8242 | 0.3425, *P*=0.1310 | 0.2070**, *P*=0.0433** | 0.3275, *P*=0.1189 |
| c.1509+32A>G | Intron 11 |  | rs144973109 | 0.8242 | 0.2528 *P*=0.0664 | 0.3186, *P*=0.1150 | 0.2601, *P*=0.0709 |
| c.1510-104C>T |  |  | Not in dbSNP | 0.2747 | n/a | | |
| c.1510-99G>A |  |  |  | 0.2747 | n/a | | |
| c.1510-43A>T |  |  | rs202069569 | 0.2747 | 0.0557, *P*=0.1847 | 0.0224, *P*=0.0885 | 0.0520, *P*=0.1734 |
| c.1510-4dupT |  |  | rs546524688 | 0.8242 | 0.2025, ***P*=0.0390** | 0.4517, *P*=0.2320 | 0.2305, *P*=0.0534 |
| c.1692G>A, p.A564= | Exon 12 | Synonymous | rs143117334 | 0.2747 | 0.0100, ***P*=0.0369** | 0.0064, ***P*=0.0341** | 0.0096, ***P*=0.0354** |
| c.1887G>A; p.T629= | Exon 13 | Synonymous | rs141651702 | 0.2604 | 0.1968, *P=*0.531 | 0.6914, *P=*0.529 | 0.2517, *P=*0.620 |
| c.1907+43C>T | Intron 13 | Intronic | rs11624081 | 9.3750 | 6.8902, *P=*0.055 | 7.7604, *P*=0.240 | 6.9869, *P*=0.067 |
| c.1907+105C>T |  |  | rs2275182 | 10.1648 | n/a | 22.2155, ***P*<0.0001** | |
| c.1908-77C>T |  |  | rs57681406 | 0.5495 | n/a | 3.3903, ***P*=0.0006** | |
| c.1935G>A, p.P645= | Exon 14 | Synonymous | rs61751177 | 1.0410 | 1.0253, *P*=0.801 | 1.0383, *P*>0.9999 | 1.0267, *P*=0.801 |
| c.2040+59dupT | Intron 14 | Intronic | rs141723381 | 1.3736 | n/a | 6.3937, ***P*<0.0001** | |
| c.2040+59delT |  |  |  | 0.2747 | n/a | | |
| c.2040+74C>G |  |  | Not in dbSNP | 0.2747 | n/a | | |
| c.2040+78C>G |  |  | rs184357448 | 0.2747 | n/a | 0.1829, *P*=0.4903 | |
| c.2041-91A>G |  |  | rs2297730 | 10.9890 | n/a | 18.7053, ***P*=0.0002** | |
| c.2116+60dupA | Intron 15 |  | rs138357816 | 1.3736 | n/a | 4.4453, ***P*=0.0045** | |
| c.2116+65A>T |  |  | rs187825570 | 0.8242 | n/a | 0.7062, *P*=0.7460 | |
| c.2116+98A>G |  |  | rs149537747 | 0.2604 | n/a | 0.6656, *P=*0.5260 | |
| c.2117-63A>C |  |  | rs17091823 | 0.5495 | n/a | 3.4945, ***P*=0.0004** | |
| c.2256+46T>C | Intron 16 |  | rs771888310 | 0.2747 | 0.0052, ***P*=0.0201** | n/a | 0.0052, ***P*=0.0201** |
| c.2256+54G>A |  |  | rs187360741 | 0.5495 | 0.0024, ***P*<0.0001** | 0.0032, ***P*=0.0004** | 0.0025, ***P*<0.0001** |
| c.2256+74C>T |  |  | rs573860725 | 0.2747 | n/a | 0.1593, *P*=0.4448 | |
| c.2257-26C>T |  |  | rs187698827 | 0.2747 | 0.0056, ***P*=0.0215** | 0.0064, ***P*=0.0340** | 0.0057, ***P*=0.0217** |
| c.2370G>A, p.R790= | Exon 17 | Synonymous | rs112712209 | 0.2747 | 0.1782, *P*=0.4781 | 0.8041, *P*=0.3781 | 0.2476, *P*=0.5947 |
| c.2436+66delA | Intron 17 | Intronic | rs34975585 | 7.6923 | n/a | 0.4280*, ***P*<0.0001** | |
| c.2436+66dupA |  |  |  | 10.4396 | n/a | 4.5379*, ***P*<0.0001** | |
| c.2804+62C>T | Intron 19 |  | rs117996122 | 1.0989 | n/a | 0.9345, *P*=0.5875 | |
| c.2804+66C>A |  |  | rs773917151 | 0.2747 | n/a | 0.0064**, *P*=0.0340** | |
| c.2804+337T>A |  |  | rs1428330577 | 1.041 | n/a | 0.0082, ***P*<0.0001** | |
| c.2804+223C>G |  |  | rs187283239 | 0.2604 | n/a | 0.7129, *P=*0.5320 | |
| c.2805-80A>G |  |  | Not in dbSNP | 0.2630 | n/a | | |
| c.2987+60T>C | Intron 20 |  | rs147668333 | 0.2747 | n/a | 0.1752, *P*=0.4759 | |
| c.2997T>G, p.L999= | Exon 21 | Synonymous | rs12018992 | 0.8242 | 0.4235, *P*=0.2019 | 1.6843, *P*=0.2990 | 0.5636 *P*=0.4651 |
| c.3033G>A, p.A1011= |  |  | rs8019857 | 0.5495 | 0.8904, *P*=0.7769 | 3.3922, ***P*=0.0006** | 1.1682, *P*=0.4561 |
| c.3094-95A>T | Intron 21 | Intronic | rs568488150 | 0.2747 | n/a | 0.2911, *P*>0.9999 | |
| c.3094-83A>T |  |  | rs964609968 | 0.5208 | n/a | 0.0129, ***P*=0.0020** | |
| c.3094-52C>T |  |  | rs763679229 | 0.8242 | 0.6363, *P*=0.5066 | 0.5977, *P*=0.4843 | 0.6286, *P*=0.5019 |
| c.3094-53dupT |  |  | rs371156380 | 3.2967 | 5.4612*, *P*=0.0696 | 0.1005*, ***P*<0.0001** | 4.3861*, *P*=0.3108 |
| c.3094-53delT |  |  |  | 2.7473 | 6.3533*, ***P*=0.0048** | 5.4300*, ***P*=0.0246** | 6.1682*, ***P*=0.0067** |
| c.3094-47G>T |  |  | rs1266048136 | 0.8242 | 0.0187*, ***P*<0.0001** | 0.0424*, ***P*=0.0009** | 0.0211*, ***P*<0.0001** |
| c.3094-43G>A |  |  | rs1180322178 | 0.2747 | 0.0004*, ***P*=0.0030** | n/a | 0.0004*, ***P*=0.0030** |
| c.3198T>C, p.T1066= | Exon 22 | Synonymous | rs114964211 | 0.5208 | 0.3313, *P*>0.9999 | 1.2197, *P>*0.9999 | 0.4300, *P*>0.9999 |
| c.3269+96A>G | Intron 22 | Intronic | rs188059193 | 0.2747 | n/a | 0.4839, *P*>0.9999 | |
| c.4206+8_4206+9insTTGTGTGT | Intron 24 |  | rs763704682 | 0.2747 | 0.1577*, *P*=0.4378 | 0.3337*, *P*>0.9999 | 0.1837*, *P*=0.4886 |
| c.4206+8_4206+9insTTGT |  |  |  | 0.2747 | 0.3288*, *P*>0.9999 | 0.9674*, *P*=0.2730 | 0.4234*, *P*>0.9999 |
| c.4206+7_4206+8dupTT |  |  |  | 0.5495 | 0.9642*, *P*=0.5932 | 0.8326*, *P*=0.7737 | 0.9447*, *P*=0.7813 |
| c.4206+9G>T |  |  | rs1778057 | 7.4176 | 46.6919*, ***P*<0.0001** | 6.6204*, *P*=0.5435 | 40.7564*, ***P*<0.0001** |
| c.4206+9delG |  |  | rs368080354 | 53.0220 | 1.0072*, ***P*<0.0001** | 19.2976*, ***P*<0.0001** | 3.2705*, ***P*<0.0001** |
| c.4206+15delG |  |  | rs778075402 | 12.3626 | 3.5930*, ***P*<0.0001** | 1.7767*, ***P*<0.0001** | 3.3677*, **P<0.0001** |
| c.4206+17delG |  |  | rs746189044 | 0.5495 | 0.3773*, *P*=0.3996 | 0.5807*, *P*>0.9999 | 0.4007*, *P*=0.6603 |
| c.4206+9_4206+19delGTGTGTGTGTG |  |  | rs748368348 | 7.4176 | 5.9790*, *P*=0.4044 | 9.1845*, *P*=0.2454 | 6.3509*, *P=*0.4044 |
| c.4206+21delG |  |  | rs781195791 | 7.6923 | 5.9050*, *P*=0.1484 | 9.1978* *P*=0.3227 | 6.2878*, *P*=0.2701 |
| c.4206+23delG |  |  | rs762786263 | 0.2747 | 0.9348*, *P*=0.2737 | 0.2430*, *P*=0.5903 | 0.8540*, *P*=0.3845 |
| c.4206+37delG |  |  | rs1224326409 | 6.5934 | n/a*^†^ | | |
| c.4206+37G>A |  |  | rs575559134 | 0.8242 | 0.0383*, ***P*=0.0004** | 0.0814*, ***P*=0.0044** | 0.0443*, ***P*=0.0007** |
| c.4206+41_4206+47delGTGTGTG |  |  | rs199508452 | 0.8242 | 0.4263*, *P*=0.2045 | 0.5819*, *P*=0.4752 | 0.4456*, *P*=0.2226 |
| c.4206+39_4206+48delGTGTGTGTGT |  |  | rs71838494 | 6.8681 | 0.2849*, ***P*<0.0001** | 0.9450*, ***P*<0.0001** | 0.3237*, ***P*<0.0001** |
| c.4206+41_4206+48delGTGTGTGT |  |  |  | 6.5934 | n/a | | |
| c.4206+47_4206+48delGT |  |  |  | 40.3846 | 3.4373, ***P*<0.0001** | 7.7455, ***P*<0.0001** | 3.6908, ***P*<0.0001** |
| c.4206+47delT |  |  | Not in dbSNP | 0.2604 | n/a | | |
| c.4206+48delT |  |  | Not in dbSNP | 0.2604 | n/a | | |
| c.4206+59A>G |  |  | rs554658369 | 0.2747 | n/a | 0.0952, *P*=0.3005 | |
| c.4206+74delT |  |  | Not in dbSNP | 0.5200 | n/a | | |
| c.4680G>A, p.A1560= | Exon 25 | Synonymous | rs61729797 | 0.5495 | 0.8926, *P*=0.7771 | 3.3985, ***P*=0.0006** | 1.1708, *P=*0.4564 |
| c.5095+13C>T | Intron 25 | Intronic | rs116247322 | 0.2747 | 0.1396, *P*=0.3992 | 0.5095, *P*>0.9999 | 0.1807, *P=*0.4828 |
| c.5095+147A>G |  |  | rs184372439 | 0.2747 | n/a | 0.0573, *P*=0.1967 | |
| c.5095+420C>T |  |  | rs1251062809 | 0.2747 | n/a | 0.0032, ***P*=0.0228** | |
| c.5095+451A>G |  |  | rs17091805 | 0.5495 | n/a | 4.0817, ***P*<0.0001** | |
| c.5095+554A>C |  |  | rs17784006 | 2.1978 | n/a | 6.6236, ***P=*0.0007** | |
| c.5096-49delG |  |  | rs141740313 | 0.5495 | 0.8838, *P*=0.7765 | 3.3518, ***P*=0.0009** | 1.1611, *P*=0.4551 |
| c.5241G>A, p.S1747= | Exon 26 | Synonymous | rs114861074 | 0.2747 | 0.1388, *P*=0.3975 | 0.5388, *P*>0.9999 | 0.1832, *P*=0.4873 |
| c.5365-111_5365-110insGAGACTTGTGATCAAGAGACAG | Intron 26 | Intronic | Not in dbSNP | 0.2747 | n/a | | |
| c.5365-83A>G |  |  | rs191304237 | 0.5495 | n/a | 0.3535, *P*=0.3721 | |
| c.5365-55G>T |  |  | rs758254099 | 0.2747 | n/a | 0.0127, *P*=0.0560 | |
| c.5527+7T>A | Intron 27 |  | rs375239471 | 0.2747 | 0.0092, ***P*=0.0341** | 0.0096, ***P*=0.0451** | 0.0092, ***P*=0.0341** |
| c.5603+87T>C | Intron 28 |  | rs145140220 | 0.5495 | n/a | 1.5828, *P*=0.1363 | |
| c.*88T>A | 3'UTR |  | rs13078 | 71.1538 | n/a | 84.9713, ***P*<0.0001** | |
| c.*166A>G |  |  | rs112795042 | 0.2747 | n/a | 0.8439, *P*=0.3804 | |
| c.*433G>T |  |  | rs10144436 | 0.2747 | n/a | 2.7831, ***P*=0.0010** | |
| c.*901T>G |  |  | rs45467692 | 0.5495 | n/a | 0.0127, ***P*=0.0019** | |
| c.*1297delA |  |  | rs578164776 | 1.9231 | n/a | 0.3315*, ***P*<0.0001** | |
| c.*1297dupA |  |  |  | 1.0989 | n/a | 2.3467*, *P*=0.1574 | |
| c.*1575A>G |  |  | rs190465278 | 0.2747 | n/a | 0.3185, *P*>0.9999 | |
| c.*1630G>A |  |  | rs1187652 | 47.2527 | n/a | 97.9191, ***P*<0.0001** | |
| c.*1718_*1719delTG |  |  | rs35500699 | 3.1250 | n/a | 6.3680**, *P*=0.009** | |
| c.*2050G>C |  |  | rs182848457 | 0.5495 | n/a | 0.1275, *P*=0.0837 | |
| c.*2309A>G |  |  | rs7158095 | 0.5495 | n/a | 3.4081, ***P*=0.0006** | |
| c.*2693A>G |  |  | rs1057035 | 13.4615 | n/a | 25.8886, ***P*<0.0001** | |
| c.*3473T>C |  |  | rs3742330 | 6.8681 | n/a | 9.7018, *P*=0.0689 | |
| c.*3886C>T |  |  | rs11556997 | 0.2747 | n/a | 0.4144, *P*>0.9999 | |
| c.*4039G>A |  |  | rs112079219 | 0.5495 | n/a | 3.3760, ***P*=0.0006** | |
| c.*4233delT |  |  | n/a | 3.1250^#^ | n/a | | |

MAF, minor allele frequency; n/a, not available; UTR, untranslated region.

All variants were annotated according to NCBI GenBank reference sequences NM_030621.4 and NP_085124.2.

* Variant located in a low complexity region: annotation or quality dubious in gnomAD.

† MAF not calculated in gnomAD due to low quality data.

# Found only in NGS cohort, not covered in WES cohort.

Text in bold refers to statistically significant differences (*P*<0.05).
